# Synergistic Role of *Streptomyces* Composite Inoculants in Mitigating Wheat Drought Stress Under Field Conditions

**DOI:** 10.3390/plants14030366

**Published:** 2025-01-25

**Authors:** Hao Shan, Hongwei Wen, Jinhui Zhang, Yuzhi Wang, Lahu Lu, Yutao Liu, Bin Yang, Wei Ji

**Affiliations:** 1 Institute of Wheat Research, Shanxi Agricultural University, Linfen 041000, China; sunway88@163.com (H.S.); sxnkywhw@163.com (H.W.); zhang-jinhui@foxmail.com (J.Z.); xmswangyuzhi@163.com (Y.W.); lulh75@163.com (L.L.); 2 College of Natural Resources and Environment, Northwest A&F University, Yangling 712100, China; sxbqf2022@163.com; 3 College of Horticulture, Shanxi Agricultural University, Taigu 030801, China

**Keywords:** wheat, drought stress, *Streptomyces* composite inoculants, photosynthetic efficiency, antioxidant defense, osmotic regulation, grain yield

## Abstract

Wheat (*Triticum aestivum* L.) is a globally important staple crop; however, its growth and yield are severely limited by drought stress. This study evaluated the effects of a combined microbial inoculant, *Streptomyces pactum* Act12 and *Streptomyces rochei* D74, on wheat photosynthesis, physiological traits, and yield under drought conditions. Key physiological and yield parameters were measured during the jointing, heading, and grain-filling stages. Drought stress significantly reduced chlorophyll content, maximum photochemical efficiency of photosystem II (PSII) (Fv/Fm), and antioxidant enzyme activities, while increasing malondialdehyde (MDA) levels, leading to a notable yield decline. In contrast, inoculation with *Streptomyces* strains alleviated these adverse effects, with the combined inoculant (Act12+D74) group demonstrating the most significant improvement. Chlorophyll content increased by up to 32.60%, Fv/Fm improved by 43.07%, and antioxidant enzyme activities were enhanced, with superoxide dismutase (SOD) activity increasing by 19.32% and peroxidase (POD) activity by 75.44%. Meanwhile, MDA levels were reduced by 61.61%. The proline content in the combined inoculant group increased by 90.44% at the jointing stage and the soluble protein content increased by 60.17% at the heading stage. Furthermore, it improved the yield by 26.19% by increasing both effective spikes and grains per spike. For the first time, this study revealed the synergistic effects of Act12 and D74 in enhancing photosynthesis, strengthening antioxidant defenses, and optimizing osmotic regulation under drought conditions. These findings provide a theoretical basis for developing environmentally friendly drought management strategies and highlight the potential applications of this inoculant in sustainable agriculture.

## 1. Introduction

Wheat (*Triticum aestivum* L.) is one of the most critical staple crops worldwide, and its production stability plays a vital role in ensuring global food security [1]. However, under the intensifying impacts of climate change, drought stress has emerged as the primary environmental constraint limiting wheat yields. Drought severely disrupts wheat metabolism by inhibiting photosynthesis, reducing water and nutrient uptake efficiency, and increasing the accumulation of reactive oxygen species (ROS), ultimately leading to significant yield losses [2]. It is estimated that approximately 45% of the global arable land is affected by drought stress, and this proportion is expected to increase in the coming years [3]. Wheat requires at least 450 mm of precipitation throughout its growth cycle to meet the basic water demand for yield formation [4]. In the North China Plain, the leading wheat-producing region in China, the annual rainfall ranges from 400 to 1000 mm [5]. However, only 20–30% of this precipitation occurs during the winter wheat growing season, leaving most fields under significant drought stress in the absence of irrigation [6]. Such persistent water deficits emphasize the urgent need to develop effective and sustainable strategies for improving wheat drought tolerance under natural rainfed conditions.

Plant growth-promoting rhizobacteria (PGPR) have garnered significant attention in recent years because of their potential to enhance drought tolerance and promote crop production. PGPR are beneficial microorganisms that colonize the rhizosphere or root surfaces, improving plant water-use efficiency through the secretion of auxins (indole-3-acetic acid, IAA), exopolysaccharides (EPS), and osmotic regulatory compounds (e.g., proline, glycine, soluble sugar, and choline). By activating antioxidant enzyme systems, PGPR effectively mitigates oxidative damage caused by the accumulation of ROS, thereby enhancing plant drought resilience [7]. Additionally, PGPR alleviates drought-induced stress on root development by increasing the activity of 1-aminocyclopropane-1-carboxylate (ACC) deaminase, which reduces endogenous ethylene levels [8]. Compared to conventional drought-resistant breeding and agronomic practices, PGPR are not only cost-effective but also environmentally friendly, making them a vital tool for advancing sustainable agriculture [9].

Among the various PGPR, *Streptomyces* spp. have emerged as a prominent research focus due to their unique drought resistance mechanisms and broad adaptability. *Streptomyces* spp. significantly enhance host plant drought tolerance by producing osmolytes, polysaccharides, and plant hormones as metabolic products [10,11]. These osmolytes function as compatible solutes that stabilize cellular structures, preserve protein function, and maintain cellular water content under drought conditions. By accumulating these solutes, plants can maintain cellular turgor and metabolic activity during water deficits [8]. Notably, its strategies, such as cell wall thickening, spore formation, and complex carbon source metabolism, confer exceptional survival capabilities in arid environments, establishing it as one of the most promising drought-resistant PGPR [12]. *Streptomyces* can inhibit the activity of pathogenic bacteria by producing antibiotics, thus showing potential for controlling soil-borne plant diseases [13,14,15]. Studies have demonstrated that *Streptomyces pactum* Act12 effectively alleviates oxidative damage induced by drought stress by increasing proline content, reducing MDA levels, and enhancing antioxidant enzyme activity [16]. Additionally, *Streptomyces pactum* Act12 improves the rhizosphere microbial community structure [17] and exhibits exceptional growth-promoting properties, such as enhancing crop yield and root vigor [18,19,20]. Similarly, *Streptomyces rochei* D74 improves plant performance under drought conditions by boosting photosynthetic efficiency and osmotic regulation capacity [21]. Notably, the combined inoculant of *Streptomyces pactum* Act12 and *Streptomyces rochei* D74 exhibits pronounced synergistic effects. It not only significantly increases crop yields [22] but also greatly enhances crop survival and stress resistance by optimizing rhizosphere microbial community structures [23,24]. This synergistic interaction highlights the immense potential of *Streptomyces* combined inoculants in drought-resilient agriculture. However, research on their application in improving wheat drought tolerance remains largely unexplored.

Based on the potential of *Streptomyces* composite inoculants for drought mitigation, their synergistic effects and multifunctionality have become a key focus in stress resistance research. Studies have demonstrated that composite inoculants exhibit greater efficiency and stability in enhancing plant stress tolerance compared to single-strain inoculants. For instance, a composite inoculant of *Bacillus cereus* G2 and *Bacillus pumilus* G5 significantly improved the salt and drought tolerance of *Glycyrrhiza uralensis* seedlings by enhancing antioxidant enzyme activities, with benefits far exceeding those of single-strain inoculants [25]. Under drought stress, co-inoculation with arbuscular mycorrhizal fungi (AMF, *Glomus versiforme*) and PGPR (e.g., *Bacillus methylotrophicus*) enhances photosynthetic capacity and drought resistance in tobacco by upregulating antioxidant enzyme activity and promoting mineral nutrient metabolism [26]. Similarly, a salt-tolerant rhizosphere-promoting composite inoculant (*Paenibacillus polymyxa* SC2, *Bacillus velezensis* DSYZ, *Lactobacillus casei* DY-3, and *Saccharomyces cerevisiae* DY-4) significantly increased cotton yield under salt stress by regulating indigenous microbial communities, optimizing photosynthesis, and improving reproductive balance [27]. These findings provide critical theoretical support for the development of composite inoculants in stress-resilient agriculture, while also highlighting the potential application of *Streptomyces* composite inoculants under drought conditions.

In our previous field experiments conducted under dryland conditions, wheat plants inoculated with *Streptomyces pactum* Act12 demonstrated significantly increased proline and soluble protein content, reduced MDA levels, and enhanced activities of antioxidant enzymes, including SOD and POD. These effects effectively alleviated the oxidative damage induced by drought stress. Additionally, wheat plants inoculated with *Streptomyces pactum* Act12 showed significant improvements in chlorophyll content, photosynthetic efficiency, grain yield, and hardness [28].

Based on these findings, this study further systematically evaluates the comprehensive effects of the combined *Streptomyces* inoculant (Act12+D74) on wheat photosynthetic characteristics, physiological traits, and yield under drought stress through field experiments. We hypothesize that wheat treated with the combined inoculant will exhibit greater improvements in photosynthetic efficiency, chlorophyll content, and antioxidant enzyme activities compared to single inoculant treatments, thereby mitigating oxidative damage induced by drought stress and ultimately enhancing growth and yield. This research was conducted in the North China Plain, a region characterized by persistent drought stress, providing an ideal environment to assess the efficacy of *Streptomyces* composite inoculants. By exploring the drought resistance mechanisms of the combined inoculant, this study aims to advance its application in agricultural production and contribute a scientific foundation for addressing the challenges posed by climate change to global food security.

## 2. Results

### 2.1. Effects of Streptomyces Inoculation on Morphological Growth Parameters of Wheat Under Drought Stress

**Figure 1:**
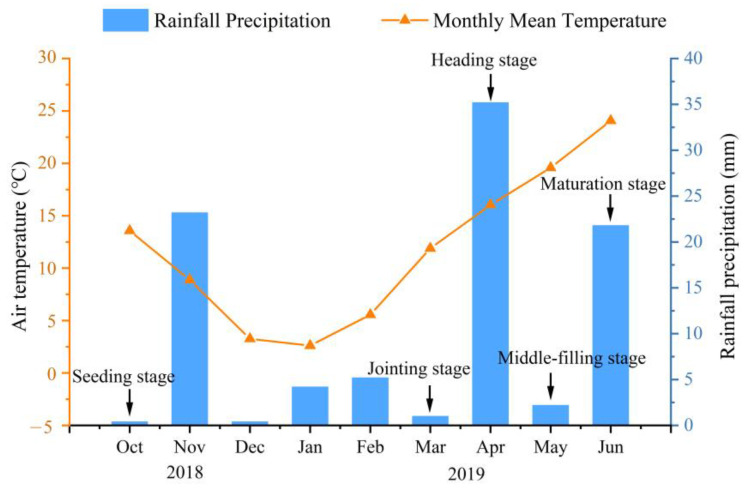
Variation in monthly rainfall and average temperature during the entire growth period of wheat.

**Figure 2:**
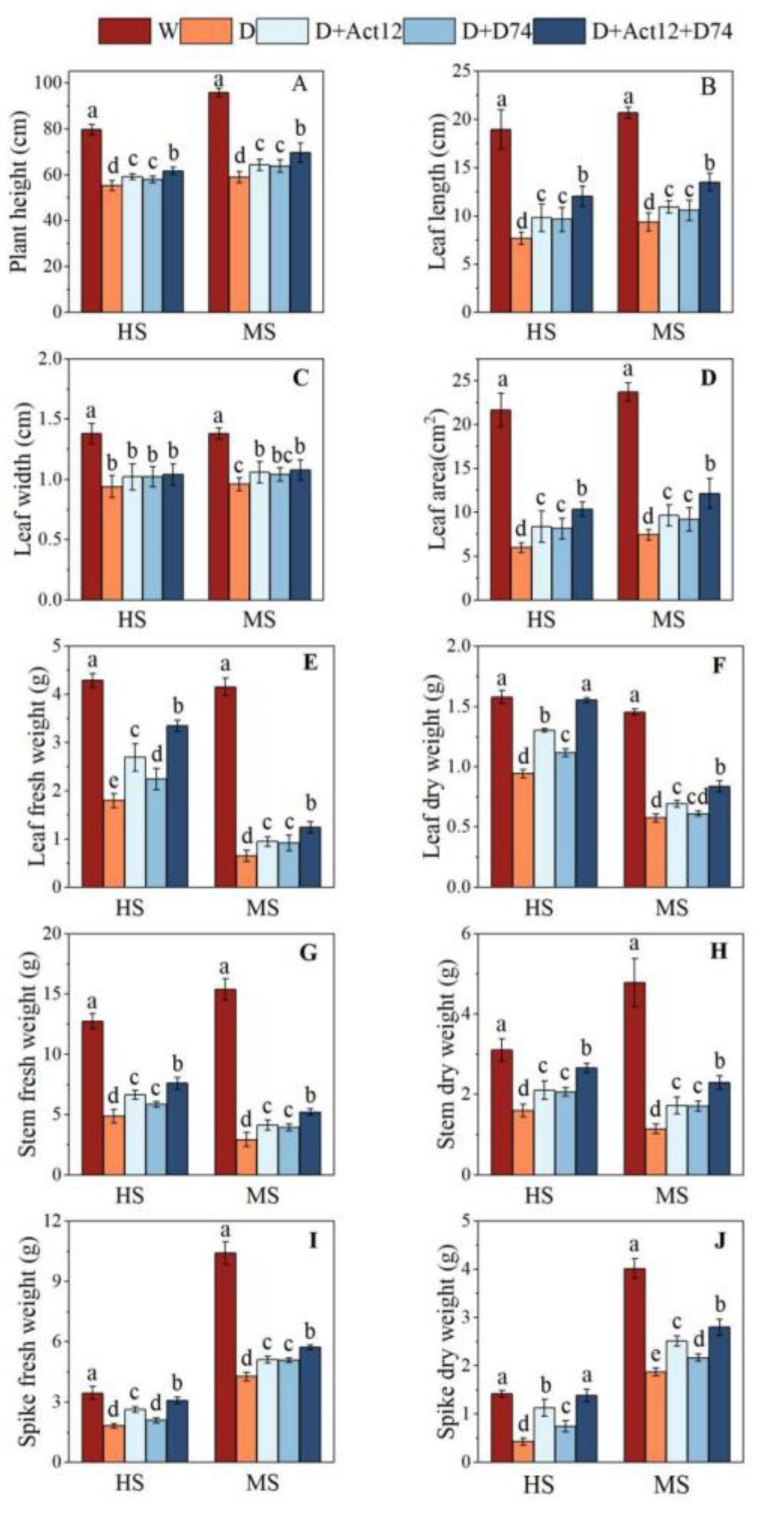
Effects of *Streptomyces* inoculation (Act12, D74, and their combination) under normal irrigation and drought stress on plant height (**A**), leaf length (**B**), leaf width (**C**), leaf area (**D**), and fresh (**E**,**G**,**I**), and dry weights (**F**,**H**,**J**) of leaves, stems, and spikes of wheat at heading (HS) and grain-filling (MS) stages. Data are presented as the mean ± SD (*n* = 5). Different letters indicate significant differences between treatments (*p* < 0.05).

Throughout the entire wheat growing season, total precipitation was only 93.6 mm (Figure 1), which is far below the water required for its growth and development. Relying solely on natural rainfall, wheat experienced drought stress throughout its growth period, especially during the jointing to heading and grain-filling stages, where water scarcity significantly hindered its growth. Drought stress significantly reduced wheat plant height, leaf length, leaf width, leaf area, and fresh and dry weights of leaves, stems, and spikes (Figure 2). Compared to the well-irrigated treatment (W group), wheat plants under drought stress (D group) showed a marked decline in morphological parameters and biomass (*p* < 0.05). However, inoculation with *Streptomyces* strains significantly mitigated these adverse effects, with the composite inoculant (D+Act12+D74) demonstrating greater effectiveness than single-strain treatments.

At the heading stage (HS) and mid-grain-filling stage (MS), inoculation with the composite inoculant increased leaf length by 57.03% and 44.44%, leaf area by 73.67% and 63.57%, and total dry weight by 88.13% and 65.06%, respectively (Figure 2B,D,F,H,J). Among the single-strain treatments, *Streptomyces pactum* Act12 exhibited superior performance compared to *Streptomyces rochei* D74, particularly in leaf fresh weight, leaf dry weight, spike fresh weight, and spike dry weight at the heading stage, as well as spike dry weight at the mid-grain-filling stage (Figure 2E,F,I,J).

### 2.2. Effects of Streptomyces Inoculation on Leaf Photosynthetic Characteristics Under Drought Stress

**Figure 3:**
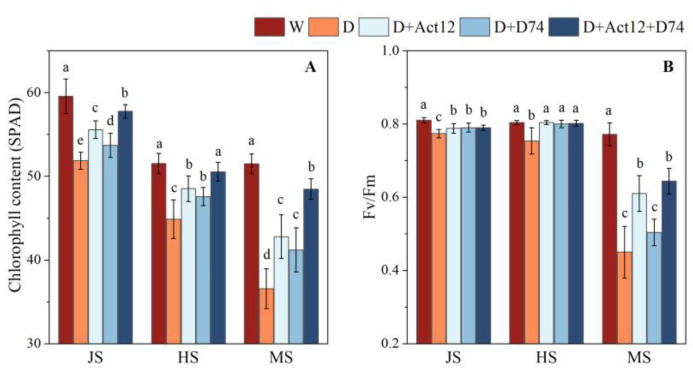
Effects of *Streptomyces* inoculation (Act12, D74, and their combination) under normal irrigation and drought stress on chlorophyll content (SPAD, (**A**)) and maximum photochemical efficiency (Fv/Fm, (**B**)) in wheat leaves at the jointing (JS), heading (HS), and grain-filling (MS) stages. Data are presented as mean ± SD (*n* = 5). Different letters indicate significant differences between treatments (*p* < 0.05).

Drought stress (D group) significantly reduced the chlorophyll content and Fv/Fm in wheat leaves (Figure 3). However, *Streptomyces* inoculation resulted in an increase in the chlorophyll content of wheat leaves, with the composite inoculant demonstrating the most pronounced improvement. Compared to the D group, chlorophyll content increased by 11.38%, 12.61%, and 32.60% at the jointing stage (JS), heading stage (HS), and mid-grain-filling stage (MS), respectively. Importantly, chlorophyll content in the inoculated plants under drought conditions was similar to that of the control group (W group), suggesting that the inoculation helped mitigate the negative effects of drought stress on the plants. While the effects of single inoculations with *Streptomyces pactum* Act12 or *Streptomyces rochei* D74 were slightly less pronounced, they still significantly slowed chlorophyll degradation in wheat (*p* < 0.05), highlighting the potential of *Streptomyces* in maintaining leaf photosynthetic capacity under drought stress.

Fv/Fm was also markedly inhibited by drought stress, with the most substantial reduction observed at the MS stage, where the D group showed a decline of 41.63% (Figure 3B). In contrast, inoculation with *Streptomyces pactum* Act12 and the composite inoculant resulted in improved Fv/Fm values, with the composite inoculant showing an 43.07% increase in Fv/Fm at the MS stage compared to the D group, indicating a notable alleviation effect. These findings indicate that *Streptomyces* inoculation effectively enhances the activity of photosystem II, mitigating the adverse effects of drought on photosynthetic efficiency.

### 2.3. Effects of Streptomyces Inoculation on Leaf Antioxidant Enzyme Activities Under Drought Stress

**Figure 4:**
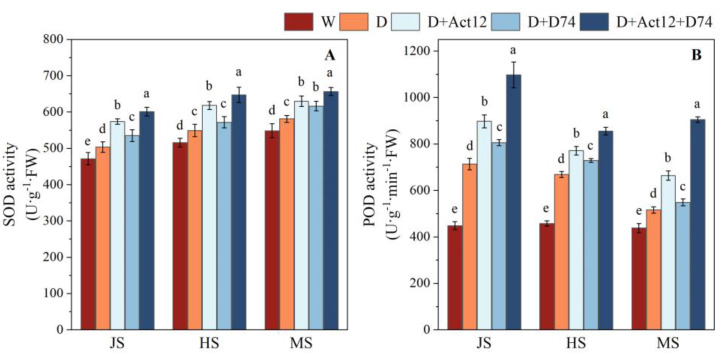
Effects of *Streptomyces* inoculation (Act12, D74, and their combination) under normal irrigation and drought stress on antioxidant enzyme activities, including superoxide dismutase (SOD, (**A**)) and peroxidase (POD, (**B**)) in wheat leaves at the jointing (JS), heading (HS), and grain-filling (MS) stages. Data are presented as the mean ± SD (*n* = 3). Different letters indicate significant differences between treatments (*p* < 0.05).

Under drought stress (D group), antioxidant enzyme activities in wheat leaves were significantly enhanced by all *Streptomyces* inoculation treatments compared with those in the well-irrigated group (W group) (*p* < 0.05) (Figure 4). Among the treatments, the composite inoculant demonstrated the most pronounced effects across all growth stages, including the jointing stage (JS), heading stage (HS), and mid-grain-filling stage (MS).

Compared to the non-inoculated drought treatment (D group), wheat plants in the composite inoculant group showed an increase in SOD activity from 12.97% to 19.32% (Figure 4A) and POD activity from 27.92% to 75.44% (Figure 4B). Additionally, wheat plants in the composite inoculant group exhibited higher enzyme activities than those in the single-strain treatments at all growth stages. These findings indicate that inoculation with the composite inoculant effectively alleviates oxidative damage caused by drought stress by significantly enhancing leaf antioxidant enzyme activities, thereby improving the drought tolerance of wheat.

### 2.4. Effects of Streptomyces Inoculation on MDA Content and Osmotic Regulatory Compounds in Wheat Under Drought Stress

**Figure 5:**
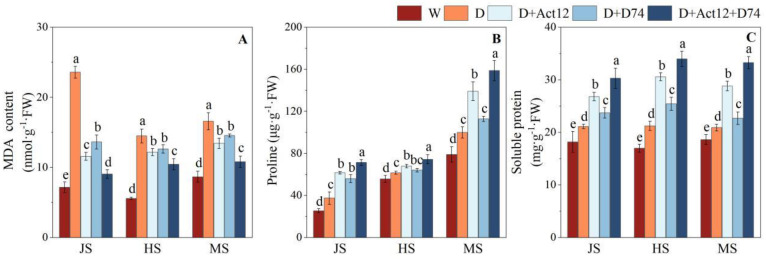
Effects of *Streptomyces* inoculation (Act12, D74, and their combination) under normal irrigation and drought stress on malondialdehyde (MDA) (**A**), proline (**B**), and soluble protein (**C**) content in wheat leaves at the jointing (JS), heading (HS), and grain-filling (MS) stages. Data are presented as mean ± SD (*n* = 3). Different letters indicate significant differences between treatments (*p* < 0.05).

Drought stress (D group) significantly increased the MDA content in wheat leaves at the jointing stage (JS), heading stage (HS), and mid-grain-filling stage (MS) compared to the well-irrigated treatment (W group) (Figure 5A). This increase reflects the extent of oxidative damage to cell membranes caused by drought stress. However, wheat plants inoculated with *Streptomyces* strains had significantly reduced MDA content compared to the non-inoculated drought treatment (*p* < 0.05). Wheat plants inoculated with *Streptomyces pactum* Act12 or *Streptomyces rochei* D74 reduced MDA levels by 15.98–50.87% and 12.23–42.23%, respectively, while the composite inoculant achieved a greater reduction of 27.89–61.61%, showing the strongest effect (Figure 5A).

Drought stress also significantly increased the accumulation of osmotic regulatory compounds, including proline and soluble proteins, in wheat leaves (Figure 5B,C). Wheat plants inoculated with *Streptomyces* strains further enhanced the levels of these compounds, with the composite inoculant showing a significantly greater effect than single-strain treatments. Specifically, the composite inoculant increased the proline content by 90.44%, 20.94%, and 58.77% in JS, HS, and MS, respectively (Figure 5B). Similarly, the soluble protein content was enhanced by 43.43%, 60.17%, and 59.11% at the corresponding stages (Figure 5C). Compared to single-strain inoculations, the composite inoculant demonstrated a significant advantage in promoting the accumulation of osmotic regulatory compounds across all growth stages. These results suggest that *Streptomyces* inoculation alleviates the adverse effects of drought stress by enhancing the accumulation of osmotic regulatory substances, thereby improving drought tolerance in wheat.

### 2.5. Effects of Streptomyces Inoculation on Wheat Yield and Related Traits Under Drought Stress

**Figure 6:**
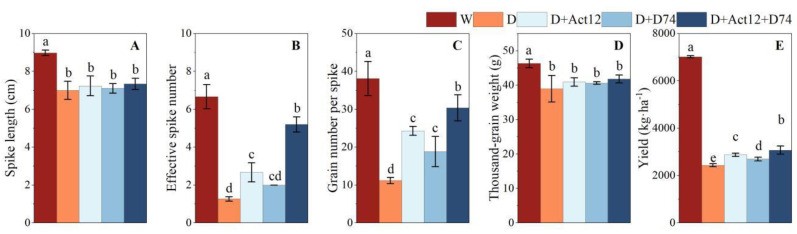
Effects of *Streptomyces* inoculation (Act12, D74, and their combination) under normal irrigation and drought stress on spike length (**A**), effective spike number (**B**), grain number per spike (**C**), thousand-grain weight (**D**), and grain yield (**E**) of wheat. Data are presented as mean ± SD; spike length, effective spike number, grain number per spike, and thousand-grain weight, *n* = 5; grain yield, *n* = 3. Different letters indicate significant differences between treatments (*p* < 0.05).

Drought stress (D group) significantly reduced wheat spike length, number of effective spikes, grains per spike, thousand-grain weight, and total yield (Figure 6). Compared to the well-irrigated treatment (W group), drought stress led to a 65.31% reduction in total yield (Figure 6E). However, wheat plants inoculated with *Streptomyces* strains showed significantly improved yield performance under drought conditions, with the composite inoculant showing the greatest effect. Specifically, compared to the non-inoculated drought treatment (D group), the composite inoculant increased wheat yield by 26.19%, while single inoculations with *Streptomyces pactum* Act12 and *Streptomyces rochei* D74 increased yield by 17.93% and 10.72%, respectively (Figure 6E).

Notably, while the effects of wheat plants inoculated with *Streptomyces* strains on spike length (Figure 6A) and thousand-grain weight (Figure 6D) were not significant, wheat plants inoculated with the composite inoculant significantly enhanced the number of effective spikes (Figure 6B) and grains per spike (Figure 6C). These improvements in yield components contributed to the substantial increase in overall wheat yield. These results suggest that *Streptomyces* inoculation, particularly with the composite inoculant, effectively mitigates the negative impacts of drought by optimizing key yield-related traits.

## 3. Discussion

Drought stress significantly limits wheat productivity, particularly in rainfed systems. It reduces photosynthetic efficiency, damages cell membranes, and increases the accumulation of ROS, all of which lead to yield losses [29]. Plant growth-promoting rhizobacteria (PGPR) have received widespread attention for their potential to enhance crop drought tolerance by modulating antioxidant defense systems and improving osmotic adjustment [30,31,32,33,34,35]. However, most studies have focused on single-strain inoculants, with limited research on the benefits of combined inoculants in improving drought tolerance in wheat under field conditions.

This study aimed to address this gap by evaluating the performance of a combined inoculant (Act12+D74) under field conditions. The results showed that the composite inoculant improved photosynthetic efficiency, antioxidant enzyme activity, and osmotic adjustment in wheat plants. These improvements led to a 26.19% increase in wheat yield under drought stress. While previous research on PGPRs, such as those using *Bacillus* spp., demonstrated their benefits in drought tolerance [30,31], our study highlights the synergistic advantages of using a combination of two *Streptomyces* strains. These findings suggest that the combined inoculant has synergistic effects, offering a potential eco-friendly solution for improving wheat production in drought-prone regions.

### 3.1. Streptomyces Enhances Wheat Drought Tolerance Through Photosynthesis and Antioxidant Mechanisms

Photosynthesis is the foundation of plant productivity; however, drought stress severely limits photosynthetic efficiency in wheat by reducing chlorophyll content, suppressing Fv/Fm, and increasing ROS accumulation [36]. In this study, we observed that inoculation with *Streptomyces pactum* Act12, *Streptomyces rochei* D74, and their combined inoculant led to increased chlorophyll content and improved Fv/Fm values in wheat leaves under drought stress (Figure 3). The combined inoculant showed the most pronounced effects, suggesting a synergistic interaction between Act12 and D74 in alleviating drought-induced inhibition of photosynthesis.

Drought stress also reduces stomatal conductance and CO₂ availability, leading to electron overaccumulation in the photosynthetic electron transport chain. This triggers ROS production, causing oxidative damage to chloroplasts [37]. The Act12+D74 combined inoculant improved PSII efficiency in capturing light energy and electron transport. This improvement not only enhanced photosynthetic efficiency but also alleviated oxidative stress, supporting sustained wheat growth under drought conditions. These findings are consistent with those of Zhao et al. [27] and Akhtar et al. [38], who observed that PGPR inoculation, particularly when combined, could significantly improve photosynthetic efficiency and mitigate oxidative stress under drought conditions.

Excessive ROS accumulation is often accompanied by intensified lipid peroxidation of cell membranes, as reflected by elevated MDA levels, which indicate the severity of oxidative damage [39]. In our study, the combined inoculant treatment resulted in a reduction in MDA levels and an increase in osmolyte concentrations, such as proline and soluble proteins. Furthermore, antioxidant enzyme activities, including SOD and POD, were significantly enhanced, strengthening wheat’s ability to scavenge ROS (Figure 4 and Figure 5). These findings suggest that *Streptomyces* mitigated oxidative damage and improved drought tolerance in wheat by enhancing antioxidant enzyme activity. This is in line with similar studies by Arora et al. [40] and Akhtar et al. [38], who showed that PGPR inoculation can enhance antioxidant capacity, reduce ROS accumulation, and ultimately improve drought resilience.

This result is consistent with previous studies, which have demonstrated that PGPRs enhance drought tolerance by improving the antioxidant defense system in various crops, including rice, wheat, maize, millet, and sorghum [38,41,42,43,44]. It is well established that ROS generated under drought stress can harm plants through oxidative stress, with enzymes and redox metabolites working synergistically to detoxify ROS. However, studies such as Girma et al. [45] suggest a contrasting view, where PGPR treatment may reduce ROS production, as evidenced by decreased activities of ROS-scavenging enzymes (peroxidase, superoxide dismutase, and catalase), along with reduced mRNA AS accumulation. Similarly, Ali et al. [46] observed that the application of Act12+biochar in Zn/Pb smelter contaminated soil led to an increase in soil enzyme activities (β-glucosidase, alkaline phosphatase, and urease), but a decrease in antioxidant activity (POD, PAL, and PPO), which could imply that PGPRs may help reduce oxidative stress in plants through mechanisms other than direct ROS scavenging.

Our findings suggest that the drought-resistant effects of *Streptomyces* sp. are linked to improvements in plant physiological processes, including enhanced osmotic regulation, increased osmolyte concentrations, and alleviation of oxidative stress. These improvements contributed to the enhanced drought tolerance observed in our study. This result is consistent with the findings of Akhtar et al. [38], who also reported that a combination of PGPR strains significantly improved drought tolerance in wheat through similar physiological mechanisms.

### 3.2. Streptomyces Improves Wheat Biomass and Yield Under Drought Stress

Drought stress severely restricts wheat growth and yield formation. This study demonstrated that *Streptomyces pactum* Act12, *Streptomyces rochei* D74, and their combined inoculant significantly alleviated the adverse effects of drought stress, with the combined inoculant exhibiting pronounced synergistic effects in improving plant height, leaf area, biomass, and yield (Figure 2 and Figure 6). The combined inoculant consistently outperformed single treatments in these parameters, further validating the synergistic interaction between *Streptomyces pactum* Act12 and *Streptomyces rochei* D74. Notably, the combined inoculant significantly increased the number of effective spikes and grains per spike. Although its effect on thousand-grain weight was modest, optimizing the yield components collectively resulted in a substantial increase in the total yield (Figure 6). These results suggest that *Streptomyces* combined inoculant mitigates the detrimental effects of drought through multiple mechanisms, thereby enhancing wheat growth and yield potential. This aligns with previous findings on the role of PGPR in improving drought tolerance and biomass in various crops, including dryland wheat, potato, maize, and rice [47,48,49,50,51].

The mechanism by which *Streptomyces* enhances wheat biomass and yield may be closely related to the regulation of hormone metabolism. *Streptomyces pactum* Act12 secretes IAA, a key plant hormone that promotes cell division and elongation, thereby enhancing the growth of roots and shoots [52,53]. Accumulated IAA not only facilitates root expansion but also improves water and nutrient uptake efficiency [1]. Additionally, ACC deaminase secreted by *Streptomyces* spp. degrades 1-aminocyclopropane-1-carboxylic acid (ACC), the precursor of ethylene, thereby reducing ethylene levels in wheat. This delays senescence and enhances drought tolerance [31,53,54]. This mechanism is similar to the findings of other studies, such as those by Zafar-ul-Hye et al. [55] and Danish et al. [56], where ACC deaminase-producing PGPR strains significantly improved drought resistance by regulating ethylene levels and promoting root growth.

Beyond the direct regulation of plant hormones, *Streptomyces* spp. may promote wheat growth and yield through its effects on rhizosphere microbial communities. Previous studies have shown that *Streptomyces pactum* Act12 increases the diversity and abundance of beneficial microbes in the rhizosphere while suppressing pathogenic microorganisms, thereby optimizing the rhizosphere micro-ecosystem [22]. These rhizosphere improvements enhance nutrient use efficiency and disease resistance, thereby supporting crop growth under drought stress. Although this study did not directly examine the effects of *Streptomyces* spp. on rhizosphere microbial communities, the significant yield improvements observed with *Streptomyces pactum* Act12 and *Streptomyces rochei* D74 suggest that their beneficial effects may be attributed, at least in part, to promoting microbial balance in the rhizosphere and enhancing root development.

### 3.3. Synergistic Effects of the Composite Inoculant on Wheat Drought Tolerance

This study is the first to systematically evaluate the performance of *Streptomyces pactum* Act12 and *Streptomyces rochei* D74 combined inoculant in mitigating drought stress in field-grown dryland wheat. These results demonstrated that the combined inoculant significantly enhanced wheat drought tolerance through pronounced synergistic effects. Compared to single inoculants, the combined treatment markedly increased the chlorophyll content, Fv/Fm values, and antioxidant enzyme activities, alleviating the oxidative damage caused by drought stress. Additionally, it elevated proline and soluble protein levels in wheat leaves, enhancing osmotic regulation and maintaining cellular water balance. These findings indicate that the synergistic interaction between *Streptomyces pactum* Act12 and *Streptomyces rochei* D74 played a crucial role across multiple levels, including photosynthesis, antioxidant defense, and osmotic regulation, providing robust support for stable wheat production under dryland conditions. These results align with previous studies that have shown that PGPR combinations can similarly improve drought tolerance and photosynthesis by enhancing antioxidant defense mechanisms and osmotic regulation [56].

The synergistic mechanism of the combined inoculant may be closely linked to its comprehensive effects on regulating endogenous plant metabolism, hormonal balance, and rhizosphere micro-ecosystems [57]. *Streptomyces pactum* Act12 and *Streptomyces rochei* D74 work synergistically to modulate plant responses in photosynthesis, antioxidant defense, and osmotic adjustment [16,21], thereby significantly improving wheat’s photosynthetic efficiency and antioxidant capacity. Furthermore, the combined inoculant’s synergistic effects on IAA synthesis and ACC deaminase activity effectively reduced ethylene levels under drought stress [57,58], delaying plant senescence and optimizing root water and nutrient uptake efficiency [59]. This mechanism is consistent with findings from other studies, such as those by Zafar-ul-Hye et al. [35] and Arora et al. [40], where PGPR combinations also reduced ethylene levels, improving drought resilience.

Although this study demonstrated the significant role of *Streptomyces pactum* Act12 and *Streptomyces rochei* D74 combined inoculant in mitigating drought stress, several issues remain to be addressed. The molecular mechanisms underlying the synergistic effects of *Streptomyces pactum* Act12 and *Streptomyces rochei* D74 are not yet fully understood, particularly their specific roles within the complex metabolic networks of photosynthesis and osmotic regulation. Furthermore, the current experiments were conducted under limited soil and climate conditions, and their performance in more diverse agricultural ecosystems and long-term effects require further validation.

Future research should integrate multi-omics approaches, such as transcriptomics, metabolomics, and microbiomics, to comprehensively elucidate the molecular mechanisms of the synergistic effects between *Streptomyces pactum* Act12 and *Streptomyces rochei* D74. This includes identifying their regulatory roles within gene networks and metabolic pathways. Additionally, broader trials are needed to evaluate the combined inoculant’s applicability and stability across various climatic conditions, soil types, and crops. This study provides a theoretical basis for the application of *Streptomyces* combined inoculants in drought-resilient agriculture and lays a practical foundation for developing sustainable drought mitigation strategies.

## 4. Materials and Methods

### 4.1. Field Experiment

The field experiment was conducted during the 2018–2019 growing season at the Hancun Experimental Station of Shanxi Agricultural University, Shanxi Province, China (111°34′36″ E, 36°8′43″ N). The experimental site is characterized by a temperate continental semi-arid climate with an average annual precipitation of 457.71 mm, evaporation of 2150 mm, and an average temperature of 13.08 °C. Temperature and rainfall precipitation were measured using a remote monitoring system with meteorological data (SY-QX-X, Shengyan Electronic Technology Co., Ltd., Handan, China).

The field had been continuously planted with wheat for five years, ensuring uniform soil fertility. The soil type was calcareous cinnamon soil with a pH of 8.67, organic matter content of 15.18 g·kg^−1^, total nitrogen content of 1.12 g·kg^−1^, alkali-hydrolyzed nitrogen of 46.51 mg·kg^−1^, available phosphorus of 7.22 mg·kg^−1^, and available potassium of 122.51 mg·kg^−1^.

The experiment included five treatments: (1) W group: normal irrigation (700 m^3^·ha^−1^) applied during the overwintering, jointing, and grain-filling stages; (2) D group: wheat plants relying solely on natural rainfall throughout the growing season; (3) D+Act12 group: seeds inoculated with *Streptomyces pactum* Act12 and grown under natural rainfall conditions; (4) D+D74 group: seeds inoculated with *Streptomyces rochei* D74 and grown under natural rainfall conditions; and (5) D+Act12+D74 group: seeds inoculated with a 1:1 mixture of Act12 and D74 and grown under natural rainfall conditions. The experiment was arranged in a randomized block design with three replicates per treatment. Each plot measured 5 m × 1.3 m.

### 4.2. PGPR Strains and Seed Treatment

*Streptomyces pactum* Act12 (GenBank: MH542148) and *Streptomyces rochei* D74 (GenBank: KJ145878) were provided by the Microbial Resources Research Laboratory, College of Resources and Environment, Northwest A&F University, China. The inoculants were prepared through solid-state fermentation by incubating for 8 days at 28 °C in peat dust. The spore density was 2.6 × 10^11^ spores g^−1^. Subsequently, the cultures were air-dried, milled, and sifted through a 0.25 mm mesh. The microbial inoculants were then prepared, yielding viable cell counts of 2 × 10^8^ to 5 × 10^8^ CFU·g^−1^, as determined by the spread plate method. The wheat cultivar used in this experiment, Jinmai 47, is a widely cultivated drought-tolerant variety in northern China.

Uniform-sized, fully developed wheat seeds were selected for the experiment. A sodium carboxymethyl cellulose (CMCNa) solution (6 g·L^−1^) was used as the binding agent. A 5% (*w*/*w*) CMCNa solution was added to the wheat seeds and stirred until the entire surface was uniformly coated [28]. The powdered inoculants were then added to the seeds, and the mixture was stirred to ensure an even coating of the inoculants on each seed’s surface. The coated seeds were air-dried in a cool place before field planting. Basal fertilizers applied included 150 kg·ha^−1^ urea (CO(NH_2_)_2_), 105 kg·ha^−1^ phosphorus pentoxide (P₂O₅), and 50 kg·ha^−1^ potassium chloride (KCl).

### 4.3. Measurement of Growth Parameters

Plant growth parameters were measured at the heading stage (HS) and mid-grain-filling stage (MS). Five wheat plants with uniform growth were randomly selected from each plot. Plant height, leaf length, leaf width, and fresh weights of the leaves, stems, and spikes were recorded. The samples were pre-dried in an oven at 105 °C for 15 min, followed by drying at 80 °C to a constant weight. The dry weight of each plant organ was then determined.

### 4.4. Measurement of Chlorophyll Content and Chlorophyll Fluorescence Parameters

Chlorophyll content and chlorophyll fluorescence parameters were measured during the jointing stage, heading stage, and mid-grain-filling stage. Five uniformly growing wheat plants were randomly selected from each plot. Chlorophyll content in the flag leaves was measured using a SPAD-502 Plus chlorophyll meter (Konica-Minolta, Tokyo, Japan) following the method of Yang et al. [60]. Chlorophyll fluorescence parameters were determined using a PAM-2500 fluorometer (Waltz, Effeltrich, Germany). Before measurement, the leaves were dark-adapted for 20 min. The measurement conditions included a light intensity of 400 μmol m^−2^ s^−1^ and a saturation pulse intensity of 8000 μmol m^−2^ s^−1^. *Fv*/*Fm* was calculated using the following formula:

(1) $$\frac{Fv}{Fm}=\frac{Fm-Fo}{Fm}$$

where *Fo* is the initial fluorescence, *Fm* is the maximum fluorescence, and *Fv* is the variable fluorescence.

### 4.5. Measurement of Antioxidant Enzyme Activities, MDA Content, and Osmotic Regulatory Compounds

Samples were collected from the entire topmost leaves of five plants at the jointing, heading, and mid-grain-filling stages. During the jointing and grain-filling stages, the topmost leaves were considered as the flag leaves. Samples were flash-frozen in liquid nitrogen and stored at −80 °C until analysis. For each treatment, the collected samples were homogenized and pooled, and three replicates were prepared for analysis.

#### 4.5.1. SOD and POD Activities

The activities of SOD and POD were measured following the method described by Tang et al. [61]. Leaf samples (0.5 g) were ground in 50 mM potassium phosphate buffer (pH 7.8) using an ice-cold mortar. The homogenate was centrifuged at 10,000× *g* for 20 min at 4 °C to obtain the enzyme extract. The activity of the SOD enzyme was determined by the reduction of nitroblue tetrazolium (NBT). The 3 mL reaction mixture contained 50 mmol L^−1^ potassium phosphate buffer (pH 7.8), 13 mmol L^−1^ Met, 75 μmol L^−1^ NBT, 2 μmol L^−1^ riboflavin, 0.1 mmol L^−1^ EDTA, and 100 μL enzyme extract. The reaction mixture was then exposed to light for a period of 20 min, with an optical intensity of 4000 lx being maintained throughout the process. The absorbance was measured at 560 nm, and the non-irradiated reaction mixture was used as a control. One unit of SOD activity was defined as the amount of enzyme required to inhibit 50% of NBT photoreduction.

Total POD activity was determined using the guaiacol method at 25 °C. The reaction mixture contained 0.1 mL of plant enzyme extract, 4 mL of 0.05 mol L^−l^ phosphate buffer, 2.0 mL of 0.05 mol L^−1^ guaiacol, and 0.1 mL of 2% H_2_O_2_. Incubate at 37 °C for 15 min, add 1.0 mL 20% trichloroacetic acid to stop the reaction, and record the change in absorbance at 470 nm within 1 min as one POD enzyme activity unit.

#### 4.5.2. MDA Content

MDA content was determined using the thiobarbituric acid (TBA) method described by Heath and Packer [62]. Leaf samples (0.2 g) were ground with 2 mL of 1% trichloroacetic acid (TCA), and the homogenate was centrifuged at 10,000× *g* for 15 min. The supernatant (1 mL) was mixed with 2 mL of 20% TCA containing 0.5% TBA and incubated in a water bath at 95 °C for 30 min. The mixture was rapidly cooled on ice and centrifuged at 10,000× *g* for 5 min. Absorbance was measured at 450 nm, 532 nm, and 600 nm to determine MDA content.

#### 4.5.3. Proline Content

Proline content was quantified using the ninhydrin colorimetric method described by Bates et al. [63]. Leaf samples (0.5 g) were extracted with 10 mL of 3% sulfosalicylic acid in a boiling water bath for 15 min, followed by centrifugation at 7000 rpm for 15 min. The extract (2 mL) was mixed with 2 mL glacial acetic acid and 2 mL ninhydrin reagent, incubated in a water bath at 100 °C for 30 min, and rapidly cooled. The reaction mixture was extracted with 5 mL of toluene, and the absorbance of the toluene phase was measured at 520 nm. Proline content was calculated using a standard curve and expressed as μg·g⁻¹ fresh weight.

#### 4.5.4. Protein Content

Protein content was determined using the Bradford method [64] with bovine serum albumin (BSA) as the standard. First, 0.2 g of leaf sample was homogenized in 10 mL of distilled water. The mixture was then subjected to centrifugation at 5000× *g* for 10 min, and the supernatant was the protein extraction solution. Subsequently, 0.1 mL of the protein extraction solution was added to 0.9 mL of distilled water and 5 mL of Comassie Brilliant Blue G-250 reagent. The mixture was then thoroughly agitated and allowed to incubate for 2 min, and the absorbance at 595 nm was measured. The protein content was calculated using a standard curve plotted against a series of BSA concentrations.

### 4.6. Measurement of Yield-Related Traits

After wheat maturity, five uniformly growing plants were randomly selected from each plot to measure spike length, number of effective spikes, grains per spike, and thousand-grain weight. Additionally, the total aboveground biomass from each plot was harvested to determine the yield.

### 4.7. Statistical Analysis

All data were analyzed using SPSS 20.0 software (SPSS Inc., Chicago, IL, USA) with the least significant difference (LSD) test at a significance level of *p* < 0.05. Graphs were prepared using Origin 2018 software (OriginPro 2018 v9.5.1.195).

## 5. Conclusions

This study highlights the significant potential of *Streptomyces pactum* Act12 and *Streptomyces rochei* D74 combined inoculant in enhancing wheat drought tolerance and yield. The combined inoculant effectively mitigated the adverse effects of drought stress by improving physiological traits, including chlorophyll content, photochemical efficiency (Fv/Fm), antioxidant enzyme activities (SOD and POD), and osmotic regulatory substances, such as proline and soluble proteins. These improvements significantly reduced oxidative damage and lowered lipid peroxidation levels, thereby alleviating the detrimental impacts of drought on wheat. Moreover, wheat plants treated with the combined inoculant demonstrated remarkable advantages in yield-related traits, promoting an increase in the number of effective spikes and grains per spike, which collectively led to a substantial improvement in wheat grain yield. *Streptomyces pactum* Act12 and *Streptomyces rochei* D74 likely exert their effects through the synergistic regulation of stress-responsive metabolic pathways, which enhance root and shoot growth, maintain hormonal balance, and optimize the rhizosphere microbial community dynamics.

Future research should focus on elucidating the molecular mechanisms underlying the synergistic effects of *Streptomyces pactum* Act12 and *Streptomyces rochei* D74 using multi-omics approaches, particularly their regulatory roles in key pathways, such as photosynthesis, antioxidant defense, osmotic regulation, and hormonal signaling. Field trials under diverse climatic conditions and soil types are also necessary to validate their applicability and stability. This study provides a robust theoretical foundation for the application of *Streptomyces* combined inoculants in drought-resilient agriculture and lays the groundwork for developing sustainable drought mitigation strategies.

## Data Availability

Data are contained within the article.
